# Value of Measuring Bone Microarchitecture in Fracture Discrimination in Older Women with Recent Hip Fracture: A Case-control Study with HR-pQCT

**DOI:** 10.1038/srep34185

**Published:** 2016-09-27

**Authors:** Tracy Y. Zhu, Vivian W. Y. Hung, Wing-Hoi Cheung, Jack C. Y. Cheng, Ling Qin, Kwok-Sui Leung

**Affiliations:** 1 Bone Quality and Health Centre, Department of Orthopaedics and Traumatology, The Chinese University of Hong Kong, Hong Kong SAR, China; 2 Department of Orthopaedics and Traumatology, The Chinese University of Hong Kong, Hong Kong SAR, China

## Abstract

We aimed to determine whether loss of volumetric bone mineral density (vBMD) and deterioration of microarchitecture imaged by high-resolution peripheral quantitative computed tomography at the distal radius/tibia provided additional information in fracture discrimination in postmenopausal women with recent hip fracture. This case-control study involved 24 postmenopausal Chinese women with unilateral femoral neck fracture (average [SD] age: 79.6[5.6]) and 24 age-matched women without any history of fracture. Each SD decrease in T-score at femoral neck (FN) was associated with a higher fracture risk (odds ratio: 6.905, p = 0.001). At the distal radius, fracture women had significantly lower total vBMD (−17.5%), fewer (−20.3%) and more unevenly spaced (81.4%) trabeculae, and thinner cortices (−14.0%) (all p < 0.05). At the distal tibia, vBMD was on average −4.7% (cortical) to −25.4% (total) lower, trabecular microarchitecture was on average −19.8% (number) to 102% (inhomogeneity) inferior, cortices were thinner (−21.1%) and more porous (18.2%) (all p < 0.05). Adding parameters of vBMD and microarchitecture in multivariate models did not offer additional discriminative capacity of fracture status compared with using T-score at FN. In old postmenopausal women with already excessive loss of bone mass, measuring bone microarchitecture may provide limited added value to improve identification of risk of femoral neck fracture.

Hip fracture is the most devastating consequence of osteoporosis in the elderly and results in more disability and consumption of healthcare resources than all other types of fracture^1^. It is associated with a reduction from expected survival of 10–20% and a downward spiral in physical functioning and quality-of-life^2^. As the world’s population ages and the number of hip fracture continues to rise^3^, finding a strategy to effectively identify individuals who are at risk of fracture and who will benefit from preventative care is key to alleviate the burden of osteoporotic fracture.

In practice, risk of hip fracture is traditionally evaluated by areal bone mineral density (aBMD) using dual-energy x-ray absorptiometry (DXA). Low aBMD is well recognized as a major risk factor for fracture. However, the predictive capacity of aBMD alone is not adequate to capture all fracture risks^4^,^5^,^6^. Methods have been developed to measure features pertinent to bone strength, such as bone geometric and microarchitecture and to incorporate these features in fracture prediction. *In vivo* quantification of architectural deterioration of the bone has become possible by using high-resolution peripheral quantitative computed tomography (HR-pQCT) at the extremities (distal radius and tibia)^7^. Previous case-control studies in Caucasian featuring HR-pQCT have revealed low volumetric BMD (vBMD) and significant deterioration of bone architecture that are independent of aBMD assessed by DXA in postmenopausal women^7^,^8^,^9^,^10^,^11^,^12^ and older men with all types of fracture or wrist fracture^13^,^14^. The discriminative capacity depended on the skeletal sites (distal radius only^11^,^15^,^16^, distal tibia only^14^,^17^,^18^, or both sites^8^,^9^,^12^,^13^), the compartments (cortical^11^,^13^,^14^,^17^, trabecular^10^,^16^,^19^, or both compartments^8^,^9^,^12^,^15^,^20^), the type of fracture (all fragility fracture^8^,^9^,^13^,^14^,^18^,^21^, wrist fracture^10^,^11^,^15^,^19^,^20^, or vertebral fracture^16^), and the severity of fracture^12^,^16^.

Most of these previous studies included patients with all types of osteoporotic fracture or patients with wrist fracture, who have a relatively wide range of aBMD. Number of patients with hip fracture is often small. It remains to be determined whether the microarchitectural deterioration revealed by HR-pQCT can have significant additional discriminative value, independent of aBMD or beyond that is provided by aBMD, in patients with hip fracture, who are usually at an advanced age and have already had substantial loss of bone mass, and in whom aBMD predicts fracture with higher gradients of risk than other ages^22^. In this case-control study, we aimed to determine the magnitude of loss of vBMD and deterioration of microarchitecture at the distal radius and tibia in postmenopausal women with recent hip fracture at the femoral neck and to explore if such deteriorations provided additional information in fracture discrimination, independently of aBMD or beyond that was provided by aBMD.

## Results

### Characteristics of participants

For this cross-sectional study, 24 consecutive postmenopausal Chinese women aged over 65 years old with recent unilateral fracture at the femoral neck were age-matched with a group of women who had never had a history of fracture and who were ambulatory. Table 1 shows the characteristics of study participants. The cohort consisted of elderly women in their late 70s. Age of the cohort ranged from 67 to 89 years and only two women were in their 60s. Fracture women and controls did not differ significantly in terms of age and body weight. Body height was slightly lower in fracture women (mean height: 1.46 vs. 1.50 m), but the difference did not reach statistical significance (p = 0.065). BMI did not differ between the two groups. Twelve patients had femoral neck fracture on the left hip and 12 on the right hip. Slip and fall was the main cause of fracture. Only one patient reported no obvious trauma that precipitated the fracture. Before the fracture event, ten women were fully ambulatory, 11 were ambulatory with crutch, and three women walked with frame.

aBMD of the femoral neck of the non-fracture hip (fracture women), or the left femoral neck (controls), and lumbar spine (L2-4) was measured by a standard DXA equipment and T-scores were calculated with reference to a local population norm. At the lumbar spine, 8 (33.3%) fracture women and 13 (54.2%) controls were categorized as osteoporotic (T-score ≤−2.5). The corresponding figures for osteopenic were 10 (41.7%) and 5 (20.8%), respectively. T-score at lumbar spine was comparable between groups (−1.9 vs. −2.2, p = 0.517). At the femoral neck, 23 (95.8%) fracture women and 12 (50%) controls were categorized as osteoporotic. The corresponding figures for osteopenic (−2.5 < T-score <−1) were 1 (4.2%) and 10 (41.7%), respectively. T-score at the femoral neck was significantly lower in fracture women than in controls (−3.8 vs. −2.3, p < 0.0001). Each unit decrease in T-score at the femoral neck was associated with a higher risk of fracture (crude OR: 6.905, 95% confidence interval [CI]: 2.224–21.44, p = 0.001) and this association was independent of age and body mass index (BMI) (adjusted p = 0.003).

### Differences in vBMD, microarchitecture, and estimated bone strength

**Figure 1:**
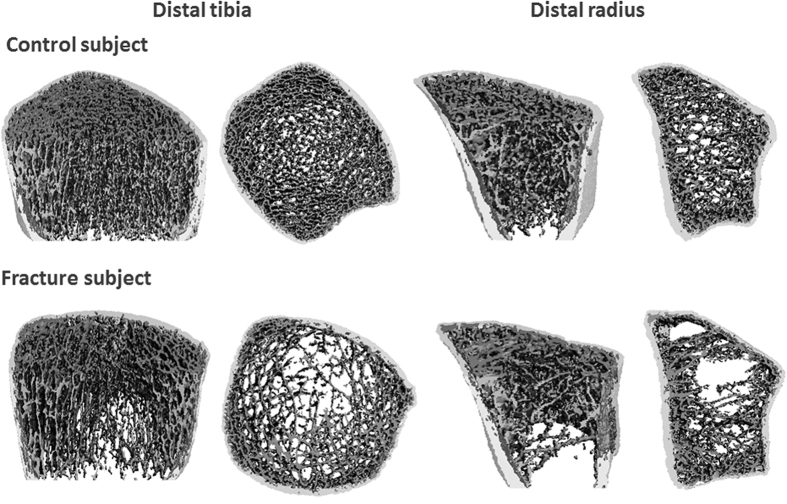
Representative 3D images of the distal radius and tibia of a femoral neck fracture women and a control. Disruption of the trabecular network is particularly noticeable in the fracture women.

Parameters of vBMD and microarchitecture were obtained from images of the distal radius and tibia from HR-pQCT. Micro-finite element (μFE) analyses were performed on images to obtained parameters of estimated bone strength. Table 2 shows differences in vBMD, microarchitecture, and estimated bone strength between the two groups. Figure 1 shows representative images of the distal radius and tibia of one fracture women and one control. At the distal radius, fracture women had significantly lower cortical area fraction (by −15.4%), lower total vBMD (by −17.5%), fewer (lower trabecular number [Tb.N] by −20.3%) and more unevenly spaced (higher inhomogeneity by 81.4%) trabeculae, and thinner cortices (lower cortical thickness [Ct.Th] by −14.0%). Differences in trabecular and cortical vBMD, trabecular thickness (Tb.Th), trabecular separation (Tb.Sp), and cortical porosity (Ct.Po) were not significant. Whole bone stiffness and failure load were significantly lower by −12.5% and −12.7%, respectively, in fracture women.

At the distal tibia, deteriorations in vBMD and microarchitecture in fracture women were more pronounced. Significant between-group differences were found for all parameters but Tb.Th (by −5.1%, p = 0.419). Compared with controls, total area was significantly higher by 10.7%, and vBMD was on average −4.7% (cortical vBMD) to −25.4% (total vBMD) lower in fracture women. Trabecular microarchitecture was on average −19.8% (Tb.N) to 102% (inhomogeneity) inferior in fracture women. Cortices were thinner (lower cortical area fraction by −23.3% and lower Ct.Th by −21.1%, both p < 0.05) and more porous (higher Ct.Po by 18.2%, p < 0.05) in fracture women than in controls. Whole bone stiffness and failure load were significantly lower by −16.9% and −16.5%, respectively, in fracture women.

### Crude and adjusted odds ratios (ORs)

Table 3 shows crude ORs of fracture status for per SD change of parameters of vBMD, microarchitecture, and estimated bone strength. Results from crude ORs were, in general, consistent with those from comparisons between the two groups, except that per SD change of cortical area fraction and Tb.N at the distal radius, and per SD change of cortical vBMD and Ct.Po at the distal tibia was not found to be associated with higher risk of fracture.

Multivariate logistic regression analyses were performed to consider the effects of other factors known to affect fracture risk, including age, BMI, and T-score at the femoral neck, and to calculate adjusted ORs for each parameter (Table 3). Adjustment of age and BMI negated some, but not all, associations between these parameters and fracture status. At the distal radius, after adjusted by age and BMI, per SD change of Tb.N, Tb.Sp, inhomogeneity, and whole bone stiffness and failure load remained significantly associated with higher risk of fracture. At the distal radius, per SD change of total vBMD, trabecular vBMD, Tb.Sp, inhomogeneity, and whole bone stiffness and failure load remained significantly associated with higher risk of fracture. When adjusted by age, BMI, and T-score at the femoral neck, adjusted ORs for all of these parameters became insignificant, indicating that per SD change of these parameters was not associated with higher fracture risk upon considering the effect of age, BMI, and T-score at the femoral neck.

Likelihood ratio tests were conducted between two nested regression models, one with age, BMI, and T-score at the femoral neck and one with age, BMI, T-score at the femoral neck, and each parameter obtained from HR-pQCT (Table 3). Results showed all p-values > 0.05, indicating that adding parameters of vBMD, microarchitecture, and estimated bone strength in the models did not offer additional discriminative capacity of fracture status when compared with the model using age, BMI, and T-score at the femoral neck.

## Discussion

In this study, we investigated the value of the additional information provided by HR-pQCT in fracture discrimination in a group of older women in their late 70s with recent femoral neck fracture. Although women with fracture had significantly lower vBMD, inferior bone microarchitecture, and lower estimated bone strength, which was more pronounced at the distal tibia than radius, all these differences became muted when adjusted by age, BMI, and T-score at the femoral neck. Adding these parameters into the regression models did not improve discrimination of fracture status when compared with using only age, BMI, and T-score at the femoral neck.

Our results appear to contradict with previous studies which concluded that measuring bone microarchitecture by HR-pQCT would provide additional information in identifying individuals at risk of osteoporotic fracture. Majority of these previous studies, however, were performed on Caucasian men or women with a history of all types of fragility fracture^8^,^9^,^13^,^14^,^18^,^21^, of only wrist fracture^10^,^11^,^15^, or of only vertebral fracture^12^. Anti-osteoporotic therapies were not an exclusion criterion for both fracture women and controls in majority of these studies. The number of patients with hip fracture were small (ranges from 1 to 8)^14^,^18^. Age range was wider and patients in these studies were expected to have wider range of aBMD. The study by Vico *et al*. is the only one that included a group of postmenopausal women with recent hip fracture^20^. They found that cortical thickness and other cross-sectional cortical geometry represent the greatest differences between hip fracture patients and controls, as well as between hip fracture and wrist fracture patients. However, such differences were not adjusted by aBMD or T-score at the hip. The added value of including parameters of bone microarchitecture in fracture discrimination was not examined.

In contrast, our study focused on only women with recent femoral neck fracture who had never received anti-osteoporotic therapies and had no history of medical conditions or treatments that could affect bone metabolism. Assessments in our study might reflect the physiological status of bone quality that precipitated the fracture event. Both groups of women were at an advanced age (70s and 80s) and had already had significant loss of bone mass, as reflected by low average T-scores and large of proportion of subjects being categorized as osteoporosis. Although HR-pQCT revealed that patients with femoral neck fracture were characterized by significant loss of cortical and trabecular vBMD and deterioration in microarchitecture, which were more pronounced at the weight-bearing distal tibia than at the distal radius, all these differences became insignificant after adjusted, in particular, by T-score at the femoral neck. This indicated that for patients with recent hip fracture, the effects of bone microarchitecture depended on the effect of aBMD and upon considering the discriminative capacity of T-score, the effects of these parameters diminished or were muted. More importantly, comparisons between two nested regression models showed that the inclusion of these parameters of vBMD, microarchitecture, and estimated bone strength offered no additional value in fracture discrimination, compared with using T-score at the femoral neck.

Although changes of bone microarchitecture can occur independently of changes of bone mass, measures of BMD and bone microarchitecture are often mildly to moderately correlated^23^. In older patients with hip fracture, ageing has already created a significant and excessive loss of bone. Measuring loss of bone mass may have encompassed the majority of loss of bone microarchitecture, and thus, may have reflected the largest share of bone fragility, leaving measuring bone microarchitecture deterioration limited room to improve the diagnosis^24^. Technical limitation of HR-pQCT may also contribute. Although the voxel size of HR-pQCT is 82 μm, its in-plane resolution is about 130 μm. When the bone loss is excessive, trabeculae and cortices are thin, and cortical pores are small, to an extent that is below the spatial resolution of HR-pQCT and that it precludes an accurate quantification of microarchitecture^11^,^20^. Moreover, hip fracture appears to have a more complicated pathophysiology than other types of fracture. It often involves not only skeletal factors, but also extra-skeletal factors^25^. Hip fracture is mostly precipitated by a fall, which indicates problems of balance, gait or mobility^26^,^27^. Visual impairment, compromised cognition, and environmental factors, such as slippery floors and badly fitted footwear, have also been shown to increase risk of hip fracture^28^,^29^,^30^. These extra-skeletal factors are likely independent of the bone, making the prediction of hip fracture more intricate than other types of fracture.

Nonetheless, our results cannot be interpreted as a negation of the value of measuring bone microarchitecture using HR-pQCT in assessing fracture risk, as in this study we focused on hip fracture, a particular type of fracture that occurs in women of advanced age and that probably has a more complicated pathophysiology than other types of fracture. Instead, it suggests that the gradient of fracture risk or the added value associated with the parameters measured by HR-pQCT may vary in different types of fracture or in different groups of patients. This constitutes an important but unanswered issue in establishing the role of HR-pQCT as a primary or secondary assessment for fracture risk. There may exist particular groups of individuals in which HR-pQCT could have a substantial added value in predicting fracture risk. These groups of individuals would likely be younger and/or have a relatively sufficient amount of bone mass. While in other groups, such as individuals of advanced age who have already had excessive bone loss, such added value of HR-pQCT would be limited. This hypothesis is supported by a study by Bala *et al*.^11^. In this study, 68 postmenopausal women with recent forearm fracture were matched with 70 women without a history of fracture. Cortical porosity at the distal radius was measured by StrAx 1.0 using images from HR-pQCT. Their results showed that in women with osteoporosis (T-score ≤−2.5), high cortical porosity was already captured by the significantly reduced aBMD. Thus, measuring microarchitecture did not identify more women with fracture than measuring aBMD alone. Similar findings were found in women with normal aBMD, in which measuring microarchitecture did not distinguish women with fracture from those without fracture. It was only in women with osteopenia (−2.5 < T-score <−1) that measuring cortical porosity significantly improved the identification of women with forearm fracture. Our results are in line with these findings. Determining, in which groups of patients, measures from HR-pQCT has the largest added value will be key in establishing HR-pQCT as a cost-effective case-finding strategy in the primary or secondary assessment of fracture risk.

Our study was limited by the relatively small sample size and was conducted in a single ethnicity cohort. Generalization of our results to other ethnicities or to the general population must be viewed with caution. Nonetheless, our study aimed to explore and provide a preliminary estimation of the size of effect or added effect of measuring bone microarchitecture using HR-pQCT in predicting this particular type of fracture. The relatively small adjusted ORs indicated a small effect size and that it requires a significantly large cohort to demonstrate a significant discriminative effect. According to a crude estimation by Miles and Shevlin, for three predictors, a small effect size requires a sample size of 600^31^. This should be taken account, from a clinical practice point of view, when establishing the role of HR-pQCT as a cost-effective assessment of hip fracture risk, either as a primary or a secondary assessment. Furthermore, relative contribution of peak bone mass and rate of age-related bone loss to fracture risk could not be examined in a cross-sectional design study. The effect of immobilization on the bone in this study would be minimal^32^, as fracture women were all previously fully or partially ambulatory and were assessed within four weeks of the event.

In conclusion, postmenopausal women with recent hip fracture had significantly lower vBMD, deteriorated cortical and trabecular microarchitecture, and lower estimated bone strength than women without history of fracture. These differences were more pronounced at the distal tibia than radius. However, when compared with using aBMD at the femoral neck alone, measuring bone microarchitecture or estimated bone strength did not improve identification of women with femoral neck fracture. It is possible that in older women with already excessive loss of bone, measuring bone mass would have captured the deterioration in bone microarchitecture. Measuring bone microarchitecture may provide limited added value to improve identification of risk of femoral neck fracture in these women.

## Methods

### Patients

For this cross-sectional study, 24 consecutive postmenopausal Chinese women aged over 65 years old with recent unilateral fracture at the femoral neck who were admitted at the Department of Orthopaedics & Traumatology, Prince of Wales Hospital were recruited between December 2008 and January 2010. All fractures were confirmed by review of radiographs and women were assessed for the study within four weeks after the fracture event. During this period, all fracture women had received surgery with stable post-operative status and were deemed fit for study assessments by their treating surgeons. Average duration between surgery and study assessment was 10.6 days (SD: 4.4, median 8.7 days, interquartile range: 8.5–12.0 days, range: 7.5–28 days). Anti-osteoporotic therapy had not been initiated during this period in view of the controversial effects of bisphosphonates in fracture healing. All fracture women were still under post-operative bedrest before study assessments. Each fracture woman was age-matched with a woman who had never had a history of fracture and who were ambulatory. Patients and controls were excluded if (1) they had known medical conditions or treatments that could affect bone metabolism or (2) they had received anti-osteoporotic therapies such as bisphosphonates. The study was approved by the Joint Chinese University of Hong Kong – New Territories East Cluster Clinical Research Ethics Committee (Ref. No.: CRE-2007.342-T) and all participants provided written informed consent. All study procedures were conducted in accordance to the guidelines approved by the ethics committee and the Declaration of Helsinki.

### DXA assessments

aBMD of the femoral neck of the non-fracture hip (fracture women), or the left femoral neck (controls), and lumbar spine (L2-4) was measured by standard DXA equipment (XR-36, Norland Medical Systems, Fort Atkinson, WI, USA). Our short‐term precision error of aBMD by DXA, expressed as the coefficient of variation (CV), was 2.07% at the femoral neck and 1.35% at the lumbar spine^33^. T-scores were calculated with reference to a local population norm^34^. By the definitions from World Health Organization, women were categorized as normal aBMD (T-score ≥−1), osteopenic (−2.5 < T-score <−1), or osteoporotic (T-score ≤−2.5).

### HR-pQCT assessments

The distal radius and tibia were scanned by HR-pQCT (XtremeCT, Scanco Medical AG, Brüttisellen, Switzerland)^7^. For fracture women, tibiae of the fracture side and non-dominant radii were measured. For controls, non-dominant radii and tibiae were measured. The subject’s arm or leg was immobilized in a carbon fiber cast fixed within the scanner gantry. A 2D scout view of the distal radius or tibia was used to define the region of interest. Reference line was placed at the hump of the wrist joint space of the radius and the endplate of the tibia. The first slice started 9.5 mm and 22.5 mm proximal to the reference line for the distal radius and tibia, respectively. A stack of 110 parallel slices was acquired at each site using an effective energy of 40 keV, image matrix size of 1,024 × 1,024, with a nominal voxel size of 82 μm.

Images were first analyzed using the standard manufacturer’s method^7^. In brief, the periosteal boundary was enclosed by a semi-automated hand-drawn contouring scheme and a threshold-based algorithm separated the cortical and trabecular components. From this standard analysis, we obtained total and trabecular vBMD in mg hydroxyapatite (HA)/cm^3^. Trabecular number (Tb.N, mm^−1^) was determined using ridge-extraction method as the inverse mean spacing of the 3D ridges. Tb.Th (mm) and Tb.Sp (mm) were derived according to standard histomorphometry methods. SD of Tb.Sp was used to describe trabecular inhomogeneity. A fully automated cortical compartment segmentation technique adapted from the method described by Buie *et al*. was used for the assessment of cortical compartment^35^,^36^. From this analysis, we obtained total cross-sectional area (mm^2^) and cortical area fraction (%), cortical vBMD (mg HA/cm^3^), Ct.Po (%) and Ct.Th (mm). Ct.Po was calculated as the number of void voxels in each thresholded cortex image divided by the total number of voxels in the cortex^36^. Ct.Th was a direct 3D calculation of the endosteal-periosteal distance.

### Estimated bone strength by μFE analysis

μFE analyses were performed on the 3D images of the distal radius and tibia using the FE-solver included in the built-in Image Processing Language software. A special peeling algorithm, specifying a minimum cortical thickness of 6 voxels, was used to identify cortical and trabecular bone tissue. μFE analysis was performed by converting the binary image data to a mesh of isotropic brick elements^37^. For all elements, a Poisson’s ratio of 0.3 and a Young’s modulus of 10 GPa was specified. A uniaxial compression test with a 1,000 N load was performed. Stiffness (kN/mm) was used to estimate the apparent bone strength. An estimate of failure load was calculated based on the assumption that bone failure occurs if >2% of the elements were strained beyond 0.7% strain^38^.

### Statistical analyses

Statistical analyses were performed using the IBM Statistics Package for Social Sciences (IBM SPSS Statistics 20, SPSS Inc, Chicago, IL, USA). Results are presented as mean ± SD and comparisons in age, body weight, body height, BMI, and T-scores were performed using two-sample *t*-test. Comparisons in vBMD and parameter of bone microarchitecture and estimated bone strength were performed using two-sample *t* test. Logistic regression analyses were performed to compute adjusted odds ratio (OR) with 95% confidence intervals (CI) for fracture status as per SD decrease in vBMD, microarchitecture, estimated bone strength, unless otherwise specified, adjusted by age, BMI, and T-score at femoral neck. Each regression model was then compared with the regression model with only age, BMI, and T-score at the femoral neck using likelihood ratio test. A p-value was determined from a chi-squared distribution with one degree of freedom. A p-value > 0.05 indicated that inclusion of the parameter did not offer additional discriminative capacity of fracture status in comparison to the model using only age, BMI, and T-score. All hypotheses were two-tailed and a p-value < 0.05 represented statistical significance.

## Additional Information

**How to cite this article**: Zhu, T. Y. *et al*. Value of Measuring Bone Microarchitecture in Fracture Discrimination in Older Women with Recent Hip Fracture: A Case-control Study with HR-pQCT. *Sci. Rep*. **6**, 34185; doi: 10.1038/srep34185 (2016).

## Figures and Tables

**Table 1:** Characteristics of study subjects.

Variables	Fracture women (n = 24)	Controls (n = 24)	p
Age, years	79.6 ± 5.6	78.7 ± 5.4	0.579
Body weight, kg	49.6 ± 9.2	54.9 ± 12.6	0.115
Body height, m	1.46 ± 0.08	1.50 ± 0.06	0.065
Body mass index, kg/m^2^	23.5 ± 5.0	24.1 ± 4.8	0.683
T-score at femoral neck	−3.8 ± 0.8	−2.3 ± 1.0	<0.0001*
T-score at lumbar spine	−1.9 ± 1.4	−2.2 ± 1.6	0.517

Results are show as mean ± SD.

*p < 0.01.

**Table 2:** Differences in volumetric bone mineral density, bone microarchitecture, and estimated whole bone strength between women with femoral neck fracture and controls.

Variables	Distal radius				Distal tibia			
	Fracture women (n = 24)	Controls (n = 24)	% difference	p	Fracture women (n = 24)	Controls (n = 24)	% difference	p
Total area, mm^2^	232.4 ± 46.8	221.2 ± 32.8	5.1	0.368	656.8 ± 119.8	593.2 ± 79.8	10.7	0.036*
Cortical area fraction, %	16.7 ± 5.6	19.7 ± 4.3	−15.4	0.049*	11.7 ± 4.9	15.2 ± 5.8	−23.3	0.026*
Tot vBMD, mgHA/cm^3^	202 ± 65	245 ± 54	−17.5	0.023*	159 ± 61	214 ± 55	−25.4	0.002**
Tb vBMD, mgHA/cm^3^	68 ± 37	84 ± 23	−18.5	0.095	78 ± 40	100 ± 20	−21.8	0.022*
Ct vBMD, mgHA/cm^3^	894 ± 53	924 ± 50	−3.3	0.059	790 ± 70	829 ± 63	−4.7	0.048*
Tb Number, 1/mm	0.937 ± 0.438	1.175 ± 0.319	−20.3	0.044*	0.880 ± 0.423	1.097 ± 0.294	−19.8	0.045*
Tb thickness, mm	0.061 ± 0.024	0.060 ± 0.009	1.7	0.854	0.075 ± 0.017	0.079 ± 0.017	−5.1	0.419
Tb separation, mm	1.342 ± 0.834	0.869 ± 0.327	54.3	0.061	1.425 ± 0.855	0.904 ± 0.284	57.6	0.048*
Tb inhomogeneity, mm	0.918 ± 0.719	0.506 ± 0.301	81.4	0.048*	1.282 ± 1.063	0.634 ± 0.487	102	0.021*
Ct thickness, mm	0.69 ± 0.18	0.81 ± 0.17	−14.0	0.036*	0.84 ± 0.30	1.06 ± 0.32	−21.1	0.016*
Ct porosity, %	9.55 ± 4.21	7.09 ± 2.20	34.8	0.023*	14.2 ± 4.1	12.1 ± 3.4	18.2	0.0498*
Stiffness, kN/mm	43.4 ± 8.0	49.6 ± 10.0	−12.5	0.029*	110 ± 26	133 ± 23	−16.9	0.003**
Failure load, N	2,184 ± 395	2,501 ± 504	−12.7	0.026*	5,604 ± 1268	6,708 ± 1134	−16.5	0.003**

Results are show as mean ± SD. Tot: total; vBMD: volumetric bone mineral density; HA: hydroxyapatite; Tb: trabecular; Ct: cortical.

*p < 0.05; **p < 0.01.

**Table 3:** Crude and adjusted odds ratios (95% confidence interval) for fracture status by per SD decrease of parameters of vBMD, bone microarchitecture, and estimated bone strength (unless otherwise stipulated).

Variables	Distal radius				Distal tibia			
	Crude OR (95% CI)	Adjusted OR (95% CI)*	Adjusted OR (95% CI)**	p†	Crude OR (95% CI)	Adjusted OR (95% CI)*	Adjusted OR (95% CI)**	p†
Total area‡	1.34 (0.73, 2.47)	1.06 (0.53, 2.11)	1.10 (0.46, 2.64)	0.828	2.00 (1.01, 3.95)^§^	1.76 (0.81, 3.56)	1.54 (0.63, 3.75)	0.321
Cortical area fraction	1.91 (0.99, 3.70)	1.74 (0.80, 3.75)	1.00 (0.39, 2.65)	0.998	2.11 (1.05, 4.23)^§^	2.04 (0.91, 4.58)	0.55 (0.14, 2.11)	0.366
Tot vBMD	2.15 (1.08, 4.28)^§^	2.19 (0.98, 4.89)	1.08 (0.39, 2.96)	0.889	2.84 (1.35, 5.97)^§^	2.92 (1.25, 6.80)^§^	0.96 (0.29, 3.21)	0.947
Tb vBMD	1.75 (0.89, 3.43)	1.96 (0.92, 4.19)	1.23 (0.50, 2.99)	0.658	2.14 (1.08, 4.23)^§^	2.26 (1.06, 4.80)^§^	1.36 (0.51, 3.60)	0.533
Ct vBMD	1.87 (0.96, 3.65)	1.72 (0.84, 3.55)	1.11 (0.44, 2.79)	0.833	1.86 (0.99, 3.50)	1.54 (0.77, 3.07)	0.88 (0.33, 2.35)	0.79
Tb number	1.94 (1.00, 3.78)	2.18 (1.01, 4.71)^§^	1.41 (0.55, 3.57)	0.471	1.88 (1.00, 3.54)^§^	1.96 (0.97, 3.96)	1.24 (0.47, 3.26)	0.663
Tb thickness	0.94 (0.52, 1.72)	1.33 (0.54, 3.24)	1.03 (0.35, 3.05)	0.958	1.28 (0.71, 2.30)	1.24 (0.69, 2.26)	1.19 (0.59, 2.40)	0.622
Tb separation‡	2.64 (1.06, 6.54)^§^	2.94 (1.04, 8.33)^§^	1.57 (0.49, 5.08)	0.422	2.82 (1.16, 6.82)^§^	2.91 (1.16, 7.28)^§^	1.65 (0.50, 5.38)	0.376
Tb inhomogeneity‡	2.73 (1.07, 7.01)^§^	3.17 (1.08, 9.30)^§^	1.56 (0.45, 5.37)	0.455	2.53 (1.15, 5.58)^§^	2.55 (1.14, 5.70)^§^	1.47 (0.54, 4.06)	0.432
Ct thickness	2.01 (1.02, 3.95)^§^	2.00 (0.94, 4.29)	0.99 (0.39, 2.55)	0.986	2.21 (1.11, 4.38)^§^	2.15 (0.98, 4.68)	0.57 (0.15, 2.09)	0.374
Ct porosity‡	2.45 (1.07, 5.60)^§^	2.07 (0.86, 4.97)	1.34 (0.42, 4.24)	0.615	1.85 (0.98, 3.48)	1.54 (0.75, 3.16)	1.07 (0.41, 2.80)	0.893
Stiffness	2.26 (1.04, 4.93)^§^	2.49 (1.08, 5.74)^§^	0.90 (0.29, 2.77)	0.857	2.79 (1.33, 5.86)^§^	0.65 (1.44, 9.28)^§^	0.95 (0.27, 3.30)	0.933
Failure load	2.35 (1.05, 5.25)^§^	2.54 (1.09, 5.92)^§^	0.97 (0.32, 2.95)	0.950	2.84 (1.33, 6.07)^§^	3.85 (1.47, 10.0)^§^	0.97 (0.27, 3.49)	0.960

vBMD: volumetric bone mineral density; OR: odds ratio; CI: confidence interval; Tot: total; HA: hydroxyapatite; Tb: trabecular; Ct: cortical. ^§^p < 0.05.

^*^Adjusted by age and body mass index.

^**^Adjusted by age, body mass index, and T-score at the femoral neck.

^†^p by likelihood ratio test comparing two nested models, obtained from chi-distribution with degree of freedom of one. A p-value > 0.05 indicates that inclusion of the parameter in the regression model does not offer additional discriminative capacity compared with that of the model with age, body mass index, and T-score at the femoral neck.

^‡^Results are expressed as per SD increase of the parameter.
